# Enhanced Stability of Calcium Sulfate Scaffolds with 45S5 Bioglass for Bone Repair

**DOI:** 10.3390/ma8115398

**Published:** 2015-11-06

**Authors:** Cijun Shuai, Jianhua Zhou, Ping Wu, Chengde Gao, Pei Feng, Tao Xiao, Youwen Deng, Shuping Peng

**Affiliations:** 1 State Key Laboratory of High Performance Complex Manufacturing, Central South University, Changsha 410083, China; shuai@csu.edu.cn (C.S.); zhouyx@csu.edu.cn (J.Z.); gaochengde@csu.edu.cn (C.G.); fengpei@csu.edu.cn (P.F.); 2 College of Chemistry, Xiangtan University, Xiangtan 411105, China; wuping401@gmail.com; 3 Department of Orthopedics, the Second Xiangya Hospital, Central South University, Changsha 410011, China; xiaotaocsu@gmail.com (T.X.); dengjunjiejj@csu.edu.cn (Y.D.); 4 School of Basic Medical Science, Central South University, Changsha 410078, China; 5 Hunan Provincial Tumor Hospital and the Affiliated Tumor Hospital of Xiangya School of Medicine, Central South University, Changsha 410013, China

**Keywords:** calcium sulfate, 45S5 bioglass, stability, selective laser sintering, scaffolds

## Abstract

Calcium sulfate (CaSO_4_), as a promising tissue repair material, has been applied widely due to its outstanding bioabsorbability and osteoconduction. However, fast disintegration, insufficient mechanical strength and poor bioactivity have limited its further application. In the study, CaSO_4_ scaffolds fabricated by using selective laser sintering were improved by adding 45S5 bioglass. The 45S5 bioglass enhanced stability significantly due to the bond effect of glassy phase between the CaSO_4_ grains. After immersing for four days in simulated body fluid (SBF), the specimens with 45S5 bioglass could still retain its original shape compared as opposed to specimens without 45S5 bioglass who experienced disintegration. Meanwhile, its compressive strength and fracture toughness increased by 80% and 37%, respectively. Furthermore, the apatite layer was formed on the CaSO_4_ scaffolds with 45S5 bioglass in SBF, indicating good bioactivity of the scaffolds. In addition, the scaffolds showed good ability to support the osteoblast-like cell adhesion and proliferation.

## 1. Introduction

The development of suitable bone scaffold for the repair of bone defects is an important challenge in bone tissue engineering [1,2,3,4]. Generally, the bone scaffold should be biocompatible to promote bone cell growth and scaffold-cell interactions, and it should be bioactive to bond strongly with bone tissues [5,6,7]. More importantly, the scaffold should possess proper stability in order to keep the shape in the process of bone repair [8].

Calcium sulfate (CaSO_4_) has been used extensively as bone defects fillers on account of the superb biocompatibility and osteoconductive, which can degrade and be resorbed completely by surrounding bone tissue [9,10,11,12]. However, CaSO_4_ fails to provide effective support for the defect site in the process of bone repair, due to its fast disintegration and poor mechanical strength [13]. In addition, CaSO_4_ demonstrates no bioactivity and is not able to form a chemical bond with the surrounding tissue [14,15].

45S5 bioglass, a SiO_2_-Na_2_O-CaO-P_2_O_5_ based biomaterial, possesses excellent biocompatibility and bioactivity and has close chemical composition to that of bone mineral [16,17,18,19,20]. Moreover, the 45S5 bioglass has a low melting point (~1050 °C) compared with the sintering temperature of the CaSO_4_ (~1100 °C). As it is introduced into CaSO_4_, the 45S5 bioglass can act as a binder to enhance the bond strength of CaSO_4_ particles after the sintering which can effectively reinforce the stability and mechanical strength of the CaSO_4_ matrix. Furthermore, the Si–O group is released during the degradation of the 45S5 bioglass which can offer nucleation sites of the bone-like apatite crystals to improve bioactivity of CaSO_4_ [21,22].

In the study, the 45S5 bioglass was introduced into CaSO_4_ to prepare the scaffolds with porous structure by selective laser sintering (SLS). The microstructure and composition were analyzed through scanning electron microscopy (SEM) and X-ray diffraction (XRD), respectively. The stability and degradability were evaluated by immersion in simulated body fluid (SBF). The mechanical properties were measured using compression and indentation tests. Moreover, the adhesion and proliferation of osteoblast-like cell on the scaffolds were investigated through SEM and fluorescent microscope.

## 2. Results and Discussion

### 2.1. Fabrication of the Scaffolds

**Figure 1:**
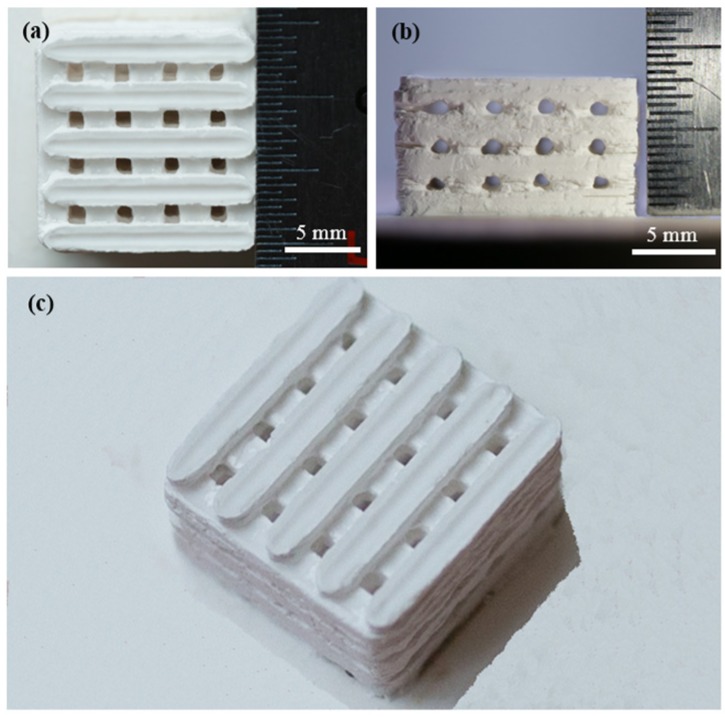
(**a**) Top view (**b**) side view and (**c**) isometric view of the CaSO_4_ scaffolds with 5 wt % 45S5 bioglass.

The scaffolds with an interconnected porous structure were obtained by SLS (Figure 1). The main preparation parameters are displayed in Table 1. The size of scaffolds was approximately 14.5 × 14.5 × 8.0 mm^3^. The pore channel was completely interconnected and branched orthogonally to form three-dimensional porosity. The wall thickness and pores size of scaffolds were about 2.0 and 1.0 mm, respectively.

**Figure 2:**
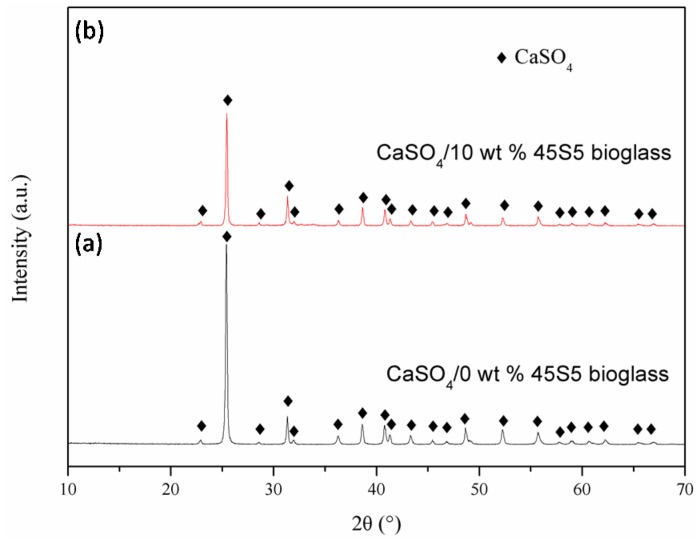
XRD patterns of the CaSO_4_ scaffolds with (**a**) 0 wt % and (**b**) 10 wt % 45S5 bioglass.

The phase composition of the scaffolds with 0, 3, 5, 10 wt % 45S5 bioglass was tested by XRD. The results showed that only the peak of CaSO_4_ was detected in all scaffolds, which revealed that the addition of 45S5 bioglass did not alter the phase composition of CaSO_4_ scaffolds and was consistent with the results of another researcher [23]. The XRD patterns of the scaffolds with 0 and 10 wt % 45S5 bioglass are displayed in Figure 2.

**Figure 3:**
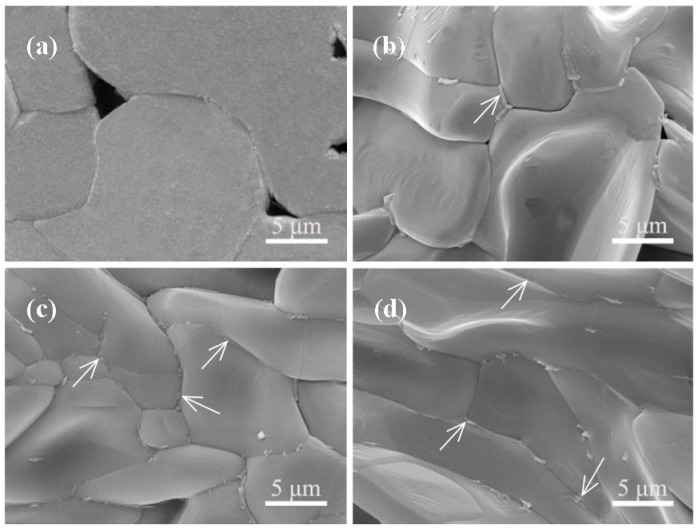
The thermally etched surface of CaSO_4_ scaffolds with different amounts of 45S5 bioglass: (**a**) 0 wt %; (**b**) 3 wt %; (**c**) 5 wt % and (**d**) 10 wt %. The arrows indicate the second phase.

The thermally etched surface of CaSO_4_ scaffolds with different amounts of 45S5 bioglass are illustrated in Figure 3. There were voids between the CaSO_4_ grains in the scaffolds without 45S5 bioglass (Figure 3a). After adding 3 wt % 45S5 bioglass, the voids reduced obviously (Figure 3b). As the amount of 45S5 bioglass increased further to 5 or 10 wt %, almost no voids occurred between the grains (Figure 3c,d). Moreover, the 45S5 bioglass as second phase was observed at the grain boundaries and more second phase appeared with 45S5 bioglass increasing (Figure 3b–d). The reason is that the melting point (~1050 °C) of the 45S5 bioglass was low compared with the sintering temperature (~1100 °C) of CaSO_4_; the bioglass melted partially during the sintering and was distributed on the CaSO_4_ grains’ boundary. In addition, the grain size of scaffolds with 45S5 bioglass was refined. This phenomenon was also observed by Kuo *et al.* who had introduced bioglass into ceramic matrix using a traditional sintering process. It could be said that the glassy phase, which lay between the CaSO_4_ grains, could be an obstacle in restraining growth of the grains [24].

**Table 1:** The preparation parameters of scaffolds.

Scan Speed (mm·min^−1^)	Laser Power (W)	Layer Thickness (mm)	Scan Spacing (mm)	Spot Diameter (mm)
100	7.0	0.1	3.0	1.0

### 2.2. Mechanical Properties

**Figure 4:**
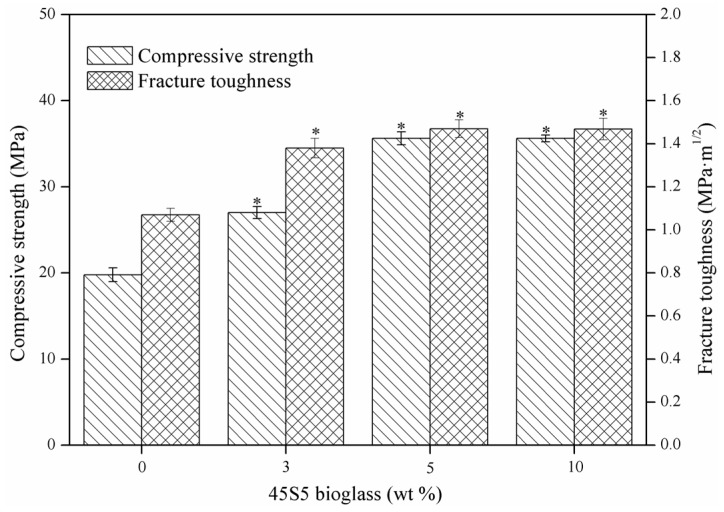
Compressive strength and fracture toughness of CaSO_4_ scaffolds with different amounts of 45S5 bioglass. Statistically significant difference (* *p* < 0.05) from scaffolds without bioglass.

Compressive strength and fracture toughness of the CaSO_4_ scaffolds with different amounts of 45S5 bioglass are displayed in Figure 4. As the 45S5 bioglass increased from 0–5 wt %, the compressive strength increased from 19.78–35.63 MPa, and fracture toughness increased from 1.07–1.47 MPa·m^1/2^. Compressive strength of the scaffolds was improved due to the bond effect of glassy phase between CaSO_4_ grains. Moreover, the glassy phase could act as a barrier to restraining the crack propagation, and, thus, enhance the fracture toughness of scaffolds.

As the 45S5 bioglass increased further to 10 wt %, no obvious difference could be found in the compressive strength and fracture toughness compared with the scaffolds with 5 wt % 45S5 bioglass. It might be ascribed to the fact that sufficient glassy phase had formed during the sintering and acted as the binder in the matrix when the amount of 45S5 bioglass reached 5 wt %. Therefore, it could be assumed that the optimal amount of the 45S5 bioglass was about 5 wt %.

### 2.3. Stability

**Figure 5:**
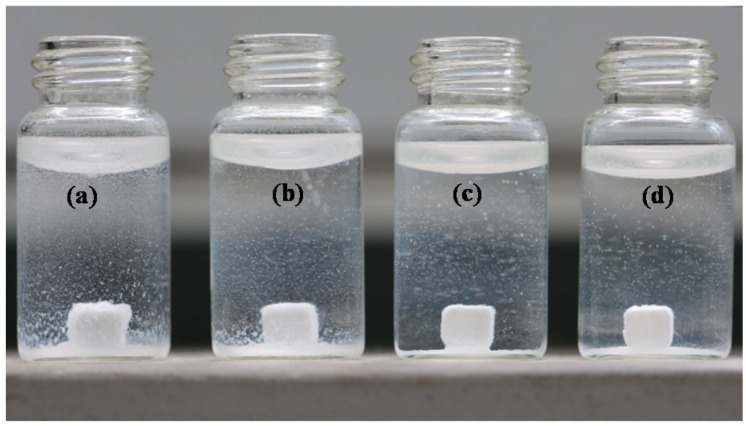
Disintegration of the specimens with different amounts of 45S5 bioglass after soaking in simulated body fluid (SBF) for four days: (**a**) 0 wt %; (**b**) 3 wt %; (**c**) 5 wt % and (**d**) 10 wt %.

**Figure 6:**
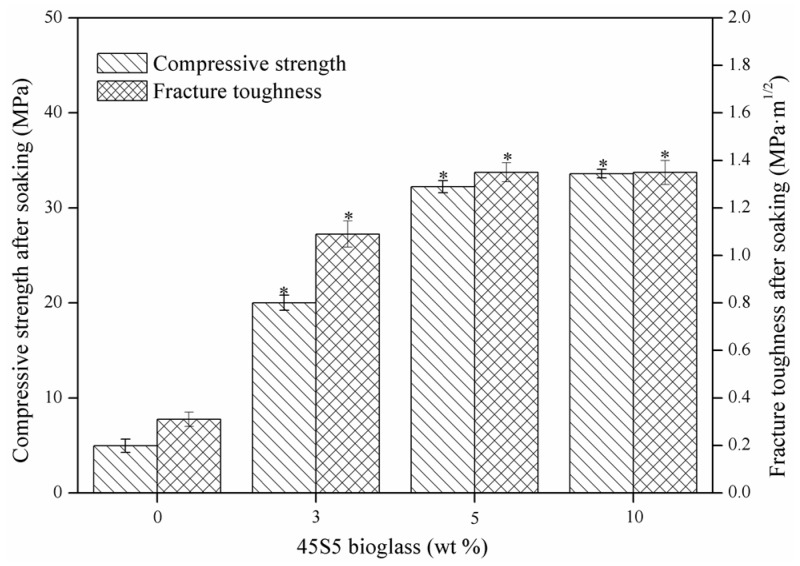
Compressive strength and fracture toughness of CaSO_4_ scaffolds with different amounts of 45S5 bioglass after soaking for four days in SBF. Statistically significant difference (* *p* < 0.05) from the specimens without bioglass.

The effect of 45S5 bioglass on the disintegration behaviour of the specimens is qualitatively shown in Figure 5. The specimens without 45S5 bioglass began to disintegrate when they were kept in the SBF for four days (Figure 5a). In contrast, only a few separated particles were observed in the surrounding solution of the specimens with 3 wt % 45S5 bioglass (Figure 5b). As the amount increased to 5 or 10 wt %, the specimens could retain their initial shape (Figure 5c,d). Compressive strength and fracture toughness of the CaSO_4_ specimens with different amounts of 45S5 bioglass after soaking for four days is shown in Figure 6. In comparison with the properties of the specimens before soaking (Figure 4), compressive strength and fracture toughness of the specimens without bioglass after soaking decreased significantly from 19.78–4.96 MPa and 1.07–0.31 MPa·m^1/2^, respectively. However, the properties of specimens with 5 or 10 wt % bioglass showed no obvious change. It suggested that the glassy phase acted as a binder between CaSO_4_ grains and could improve the stability of the specimens. This enhancement theory of stability was similar to that of gelatin enhancing calcium silicate by Wang *et al.* [25].

**Figure 7:**
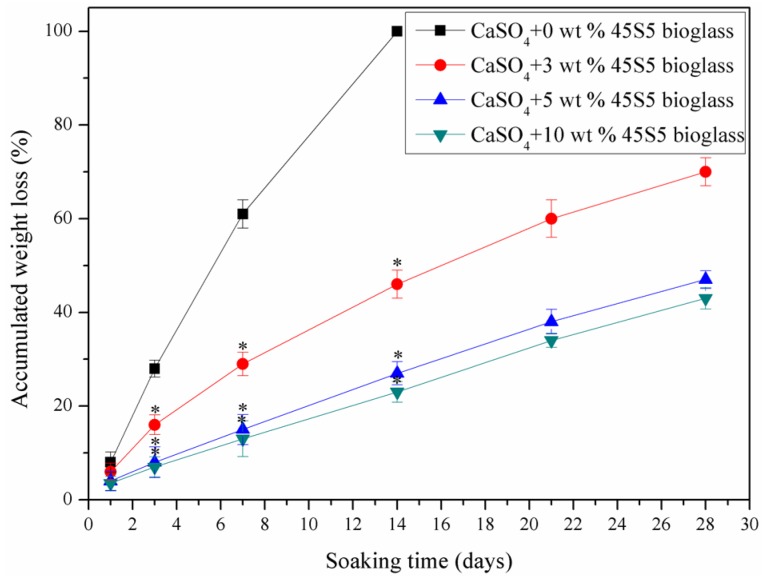
Accumulated weight loss of the specimens with different amounts of 45S5 bioglass after soaking in SBF for different durations. Statistically significant difference (* *p* < 0.05) from the specimens without bioglass.

The accumulated weight loss of the specimens with different amounts of 45S5 bioglass after soaking in SBF for different durations is shown in Figure 7. It could be seen that the specimens without 45S5 bioglass degraded completely within approximately two weeks. However, the degradation rate decreased obviously with addition of the 45S5 bioglass and slowed gradually with increases in the amount of 45S5 bioglass. The 45S5 bioglass possessed a slower dissolution rate than CaSO_4_. It was distributed on the CaSO_4_ grains’ boundary and could reduce the contact of the CaSO_4_ with the soaking solution which could decrease the degradation rate of the specimens.

### 2.4. Bioactivity

**Figure 8:**
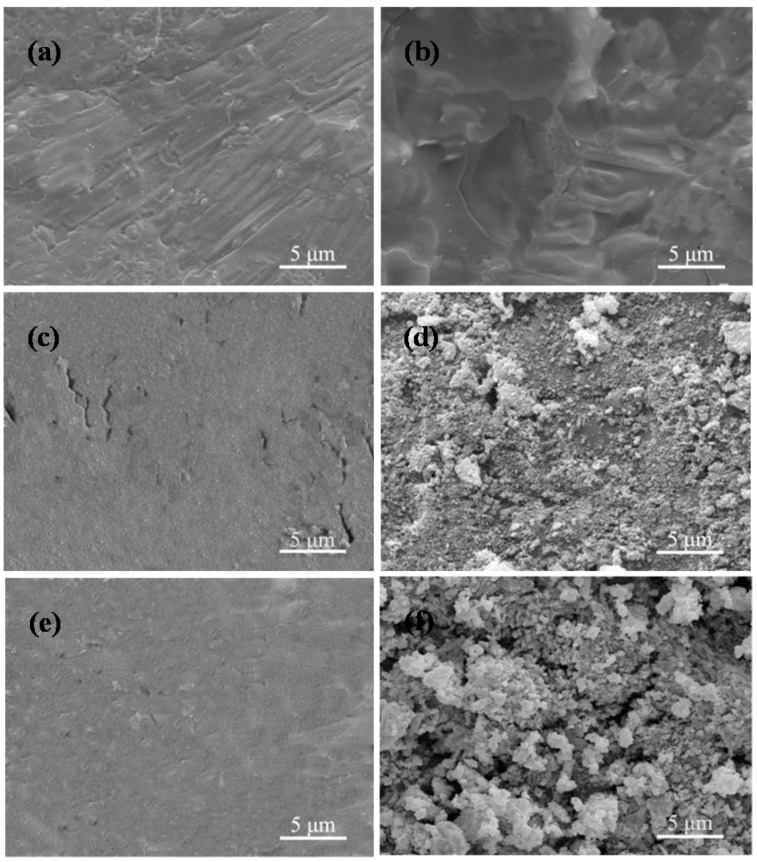
The morphological features of CaSO_4_, CaSO_4_/5 wt % 45S5 bioglass scaffolds after soaking in SBF for (**a**,**b**) zero day; (**c**,**d**) one day and (**e**,**f**) four days.

**Figure 9:**
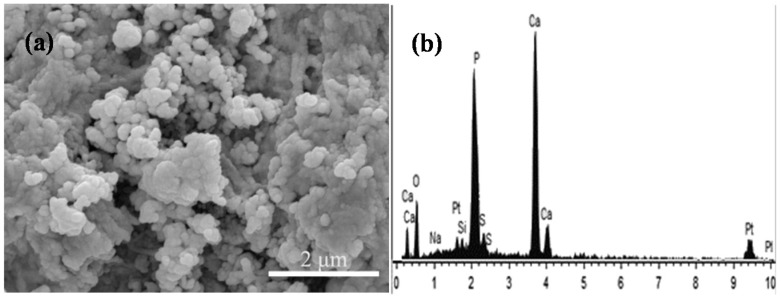
The immersion of CaSO_4_ scaffolds with 45S5 bioglass in SBF for four days: (**a**) morphology and (**b**) Energy dispersive spectroscopy (EDS) analysis. The arrow indicates the crystals for EDS analysis on the composite scaffolds.

The morphologies of the CaSO_4_ scaffolds with 0 and 5 wt % 45S5 bioglass after soaking in SBF for zero, one and four days are displayed in Figure 8. It was obviously observed that no deposition was observed on the scaffolds without 45S5 bioglass (Figure 8a,c,e). In contrast, a mass of deposition with the bone-like apatite shapes appeared on the scaffolds with 45S5 bioglass after soaking. Besides, the bone-like apatite amount notably increased with the extension of soaking time, and a layer of apatite formed on the scaffolds with 45S5 bioglass after soaking for four days (Figure 8b,d,f). By Energy dispersive spectroscopy (EDS) analysis, the Ca/P ratio of deposition was ~1.73 which was close to that of apatite (1.67), suggesting that the deposition on the scaffolds was the apatite (Figure 9).

**Figure 10:**
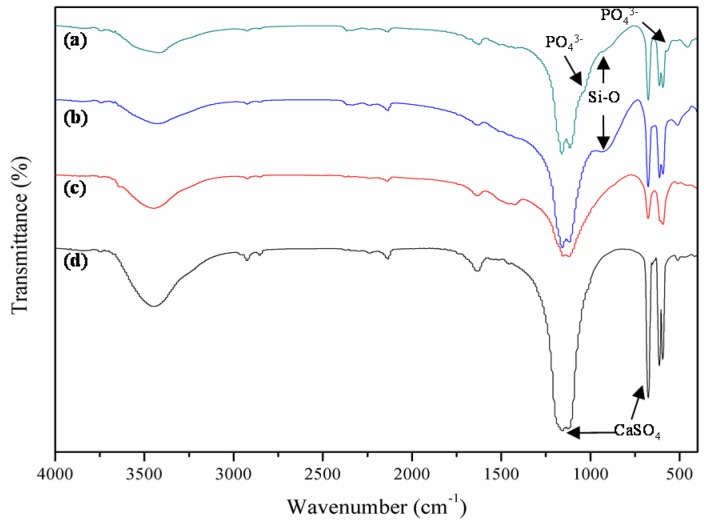
Fourier transform infrared (FTIR) spectra of the CaSO_4_ scaffolds with and without 45S5 bioglass after and before soaking in SBF: (**a**) with and (**c**) without 45S5 bioglass after soaking; (**b**) with and (**d**) without 45S5 bioglass before soaking.

The deposited apatite on the surface of scaffolds was further identified by Fourier transform infrared (FTIR) spectroscopy. The spectra of the scaffolds with and without 45S5 bioglass after and before immersing in the SBF were shown in Figure 10. The bands around 1037 and 570 cm^−1^ presented in the spectrum of the scaffolds with 45S5 bioglass after soaking (Figure 10a), which was attributed to the stretching vibration of PO_4_^3−^ group. However, these bands did not appear in other spectra (Figure 10b–d). It suggested that the apatite formed on the scaffolds with 45S5 bioglass after soaking. Furthermore, the band of Si–O group was detected at 938 cm^−1^ in the scaffolds with 45S5 bioglass.

As some previous studies *in vitro* and *in vivo* have pointed out, at the early stage of treating, CaSO_4_ could not form a chemical bond with surrounding bone tissue due to its poor bioactivity [26,27], which is also supported by the result in this study that no apatite deposition was found for the CaSO_4_ scaffolds after soaking in SBF. However, the combined SEM, FTIR and EDS analyses showed that the CaSO_4_ scaffolds with 45S5 bioglass can induce formation of the bone-like apatite within four days, indicating that the scaffolds possessed good bioactivity. After adding 45S5 bioglass, the Si–O groups were released in SBF which could provide the nucleation sites of apatite crystals [28]. Based on the obvious increased forming ability of apatite, the scaffolds with 45S5 bioglass were expected to form a firm bond with the surrounding tissue after implantation.

### 2.5. Cell Attachment and Proliferation

**Figure 11:**
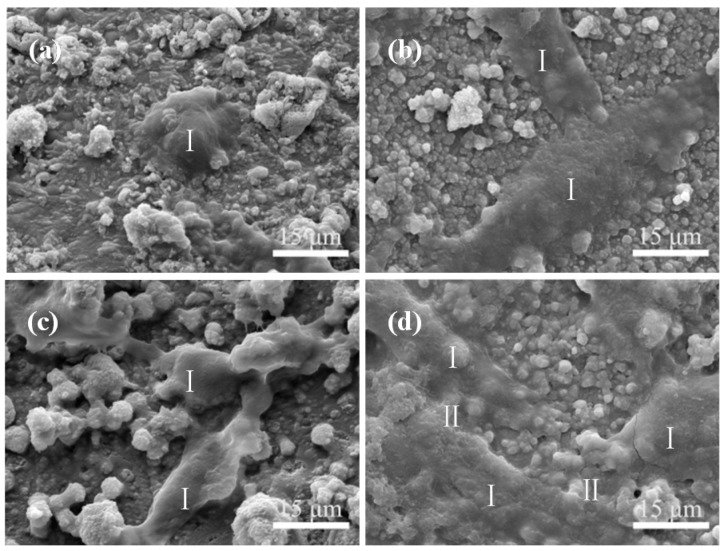
Scanning electron microscopy (SEM) images of cells cultured on the CaSO_4_ scaffolds with: (**a**) 0 wt % (control) and (**c**) 5 wt % 45S5 bioglass for one day; (**b**) 0 wt % (control) and (**d**) 5 wt % 45S5 bioglass for three days. I: cells. II: cell-cell junctions.

**Figure 12:**
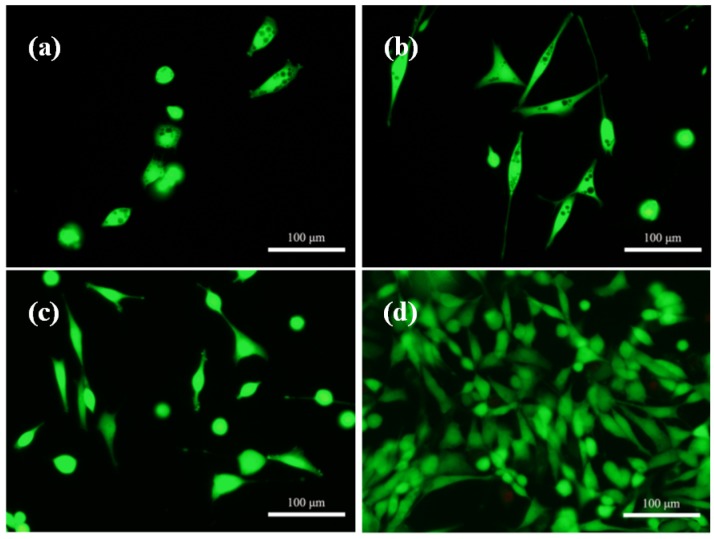
Fluorescent images of cells cultured on the CaSO_4_ scaffolds with: (**a**) 0 wt % (control) and (**c**) 5 wt % 45S5 bioglass for one day; (**b**) 0 wt % (control) and (**d**) 5 wt % 45S5 bioglass for three days.

Morphologies of the cell attachment and growth on the CaSO_4_ scaffolds with and without (control) 45S5 bioglass are displayed in Figure 11. In comparison to the round cells on the control scaffolds (Figure 11a), the cells cultured on the scaffolds with 45S5 bioglass within one day presented a spindle form and long cell extension (Figure 11c). After cultivating for three days, the cells spread well and covered most of the surface. Meanwhile, the cell-to-cell junctions by cytoplasmic extension appeared obviously on the scaffolds (Figure 11d), revealing the excellent biocompatibility of the scaffolds. It could be attributed to the adsorption of serum proteins on 45S5 bioglass and the release of ions containing Si from 45S5 bioglass. Si was an important trace element in bone formation which had a strong stimulatory effect on cells activities. The improvement of cell attachment and spreading is further shown in the Live/Dead stained fluorescent images (Figure 12).

**Figure 13:**
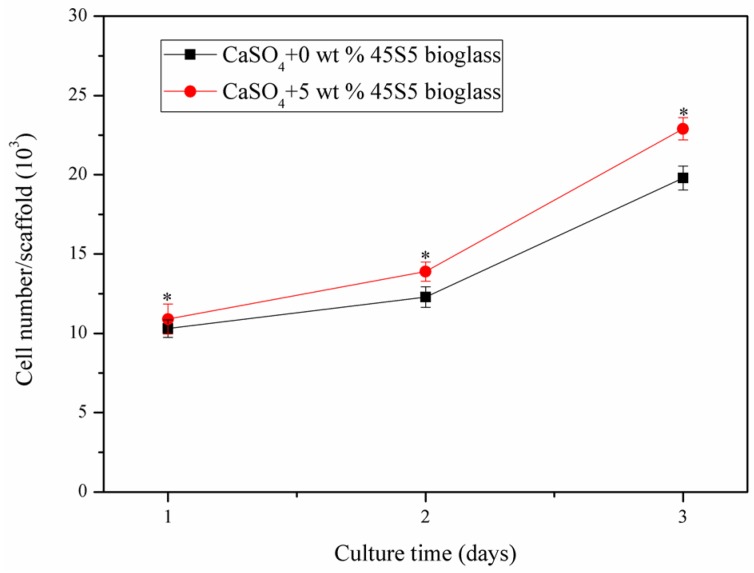
Microculture tetrazolium test (MTT) assay for proliferation of MG-63 cells on the scaffolds. Statistically significant difference (* *p* < 0.05) from the scaffolds without bioglass.

Moreover, cell proliferation was estimated by microculture tetrazolium test (MTT) assays and was displayed in a cell number curve with various periods of cell culture (Figure 13). The statistics analysis results showed that the number of cells that gradually grew on the scaffolds with the different culture times and cell proliferation rates on the scaffolds with 45S5 bioglass was improved when compared to the control group, with demonstrated that CaSO_4_ scaffolds with 45S5 bioglass were suitable for the growth and proliferation of cells. In recent years, some research verified that the bioglass could be introduced to ceramic to improve cells activity [29]. The results in this test also indicated that the CaSO_4_ scaffold with 45S5 bioglass fabricated using SLS had good biocompatibility.

## 3. Materials and Methods

### 3.1. Scaffolds Preparation

Medical-grade calcium sulfate anhydrate (CaSO_4_) was applied as the starting powder material which was obtained from Alfa Aesar China Co., Ltd. (Shanghai, China). Some relevant parameters of CaSO_4_ powder were described as follows: melting point: 1450 °C, purity: 99%, density: 2.960 g·cm^−3^. 45S5 bioglass powder (45 wt % SiO_2_, 24.5 wt % CaO, 24.5 wt % Na_2_O and 6 wt % P_2_O_5_) was purchased from Chinese Kunshan Technology New Materials Co., Ltd. (Kunshan, China). The average particle size and melting point of 45S5 bioglass powder was 45–50 μm and ~1100 °C, respectively. The 45S5 bioglass powders (3, 5 and 10 wt %) were added respectively to the CaSO_4_ powder by using a mechanical mixing method to prepare CaSO_4_/45S5 mixed powders. The mixed procedure was as follows: CaSO_4_ and appropriate amounts of 45S5 bioglass were dispersed in anhydrous alcohol and sonicated for 30 min to reduce agglomerate formation. Then, the mixed powders were milled for 10 h using the ZrO_2_ ball which acted as the milling media to enhance the homogeneity. After grinding, the mixed powders were dried in an oven at 50 °C.

The mixed powder was used to prepare porous scaffolds through selective laser sintering (SLS). SLS process was described in detail as follows: (a) The powder was paved onto the build platform to form a thin powder layer; (b) According to sliced 2D patterns, the spread powder layer was sintered selectively by a laser beam and formed a solid layer; (c) The build platform of the system is lowered to about the thickness of powder monolayer; (d) Steps (a)–(c) are repeated, and a multi-layer part is prepared by stacking sintered powder layers. Finally, the residual powder is removed and a porous scaffold was obtained.

### 3.2. Microstructure

The CaSO_4_-based scaffolds were characterized using X-ray diffraction (XRD, Bruker AXS Inc., Karlsruhe, Germany) and scanning electron microscopy (SEM, TESCAN, Brno, Czech Republic). Before XRD test, the scaffolds were ground into powder samples. The phase composition of the scaffolds were analyzed using XRD with monochromatic Cu Kα X-ray (λ = 1.54056 Å, 40 kV, 250 mA) in a continuous scan mode. The diagram was recorded in 10° ≤ 2θ ≤ 70° at a scanning speed of 8°/min. The microstructure was observed by SEM, and the images were collected by using the apparatus operating at 40 mA, 20 kV, and a working distance (WD) of 15 mm.

In addition, the change of the functional groups before and after immersion of the scaffolds in SBF were measured by FTIR with a Nicolet 6700 spectrometer (Thermo Scientific Co., Madison, WI, USA). For this, 1 mg of powder scraped from the surface of scaffolds and 250 mg of KBr (infrared (IR) grade) were mixed and pressed into a small disk which was suitable for FTIR test. Then, the disk was analyzed with a resolution of 8 cm^−1^ in 4000–400 cm^−1^ range.

### 3.3. Mechanical Test

The compressive strength of CaSO_4_-based scaffolds was tested at the maximum load of 100 N and rate of 0.5 mm·min^−1^ by using a universal testing machine (WD-01, Shanghai Zhuoji instruments Co. LTD, Shanghai, China). Six scaffolds were carried out for per group. The fracture toughness (*K*_IC_) was measured according to the indentation method. Briefly, Vickers indenter (HXD-1000TM, Shanghai Taiming Optical Instrument, Shanghai, China) printed an indentation on the center of the mirror polished surface of the scaffolds and tested six times. Crack radius was observed by the indenter microscope. The fracture toughness was obtained using the Equation (1) developed by Evans and Charles [30], which relates to the indentation load (*P*) and the radial crack length (*c*):

*K*_IC_ = 0.0824(*P*/*c*^3/2^) (1)

### 3.4. The Stability and Weight Loss

The disintegration rate of CaSO_4_-based specimens was qualitatively tested as follows: Cube-shaped specimens (4.0 × 4.0 × 2.5 mm^3^) were immersed immediately in 5 mL simulated body fluid (SBF) after sintering and left at 37 °C for 4 days. Then, the visual disintegration situation of the specimens was recorded with the digital camera (Canon 5D Mark III, Canon Inc., Tokyo, Japan). Moreover, compression and fracture properties of specimens after soaking were measured after soaking. A total of six samples were tested for each group.

The specimens were placed into SBF at 37 °C to study their degradability. The soaking solution was refreshed every 24 h. After soaking on different days, the specimens were taken away from the solution and dried at room temperature. Their weight loss was calculated using the following Equation (2) [31]:

Weight loss = (*w*_1_ − *w*_2_)/*w*_1_ (2)

where weight of the specimens before soaking was designated as *w*_1_ and weight of the specimens after soaking was designated as *w*_2_.

### 3.5. In Vitro Bioactivity

The SBF solution was prepared to test bioactivity of CaSO_4_-based scaffolds *in vitro*. The ion concentrations of the solution are similar to human blood plasma. It was obtained by dissolving the following some ions into distilled water in sequence: 142.0 mmol·L^−1^ Na^+^, 5.0 mmol·L^−1^ K^+^, 2.5 mmol·L^−1^ Ca^2+^, 1.5 mmol·L^−1^ Mg^2+^, 27.0 mmol·L^−1^ HCO_3_^−^, 1.0 mmol·L^−1^ HPO^2−^, 103.0 mmol·L^−1^ Cl^−^, 0.5 mmol·L^−1^ SO_4_^2−^. For evaluation of bioactivity of scaffolds *in vitro*, the CaSO_4_ and CaSO_4_/45S5 composite scaffolds were soaked in the SBF solution (pH = 7.4) at 37 °C for 0, 1, and 4 days without refreshing the soaking medium. The scaffolds were soaked for different times and then dried at room temperature after gently cleaning with deionized water. After that, they were taken away from the solution. The formation of bone-like apatite layer on the scaffolds was determined by SEM, EDS microanalysis and FTIR spectroscopy using KBr technology.

### 3.6. Cell Attachment and Proliferation

Cell attachment and proliferation on the CaSO_4_-based scaffolds were carried out which were cultivated in Dulbecco’s Modified Eagle’s Medium (DMEM) and were supplemented with 100 U/mL penicillin-streptomycin and 10% fetal bovine serum (FBS, American Type Culture Collection (ATCC), Rockville, MD, USA). The operational cell in the biocompatibility test was MG-63 human osteoblast-like cell which was purchased from ATCC. Before being seeded, the cells were isolated with trypsin-ethylenediaminetetraacetic acid (EDTA) and resuspended in culture medium. The scaffolds were sterilized using ultraviolet light and incubated in culture medium at 37 °C with 5% CO_2_ and 85% humidity for 1 h before seeding cells. Then, the scaffolds were removed gently from the culture medium after a certain period. The isolated cells were implanted drop wise onto the scaffolds at the concentration of 5 × 10^3^ cell/cm^2^ and fully absorbed by the media. Consequently, the scaffolds with cells were cultured in the incubator in the humidified atmosphere at 5% CO_2_ and 37 °C to allow the cell adhesion and proliferation. After cultivating for 1 and 3 days, the scaffolds were washed for half an hour with phosphate buffered saline (PBS) with 2.5% glutaraldehyde, and then thoroughly cleaned with PBS. Sequentially, the scaffolds were dehydrated with the graded alcohol series and air-dried. Finally, the scaffolds were sputtered with platinum in vacuum and used for SEM analysis.

For cell staining, the CaSO_4_-based scaffolds with cultured cells were placed in PBS with 4% paraformaldehyde for 25 min. Sequentially, the scaffolds were cleaned with PBS and soaked in PBS with 0.5% Triton X-100 for 5 min. Then, the scaffolds were soaked with 1% FBS (in PBS), cleaned with PBS, stained with calcein AM (green) along with the Live/Dead viability/cytotoxicity kit and placed in dark environment for 5 min. The scaffolds were then cleaned with PBS and observed using fluorescent microscope (Olympus, Tokyo, Japan). MTT assays were also carried out in the study to assess the proliferation of MG-63 cell on the scaffolds after cultivating for 1, 2 and 3 days. Six scaffolds were carried out for per group. The MTT solution was injected to cells culture well at different days. The formazan crystal formed on account of MTT decrease by viable cells after the scaffolds were cultivated for about 5 h. Then, the formazan crystals dissolved completely through adding dimethyl sulphoxide (DMSO) to cells culture wells after the MTT solution was taken away. In the end, the scaffolds were removed and the absorbance at wavelength of 490 nm was measured using the microplate reader. The intensity of absorbance was directly proportional to the cell number.

### 3.7. Statistical Analysis

Experimental results were indicated as mean ± standard deviation of six separate replicates. Statistical significance between the mean values was determined using one-way analysis of variance (ANOVA). The significance of the standard deviations was determined by Scheffe’s multiple comparison testing. The results were deemed statistically different for *p* < 0.05 (significance level).

## 4. Conclusions

The CaSO_4_ scaffolds with 45S5 bioglass were successfully obtained using SLS. The stability and mechanical properties of the scaffolds were improved significantly by introducing 45S5 bioglass, due to the bond effect of glassy phase between the CaSO_4_ particles. Moreover, the scaffolds demonstrated good forming ability of bone-like apatite in SBF which showed good bioactivity. The cell test indicated that the scaffolds were not only suitable for cell growth, but also stimulatory to cell proliferation. It was indicated that the CaSO_4_ scaffolds with 45S5 bioglass had significant advantages in use for bone grafts in clinical applications.
